# Evaluation of NLRP3 inflamassome expression and its endogen inhibitor CGI-58 in subjects with different obesity degrees

**DOI:** 10.1186/1758-5996-7-S1-A141

**Published:** 2015-11-11

**Authors:** Jakeline Rheinheimer, Milene Moehlecke, Natali Silva Cardoso, Mariana Lopes dos Santos, Luís Henrique Canani, Cristiane Bauermanno Leitã, Daisy Crispim

**Affiliations:** 1 Serviço de Endocrinologia-Hospital de Clínicas de Porto Alegre, Porto Alegre, Brazil

## Background

Obesity is associated with a state of low-grade chronic inflammation, commonly causing insulin resistance (IR) and type 2 diabetes mellitus (T2D). The NLRP3 inflamassome is a key mediator of metabolic inflammation, and it has been shown to be activated in macrophages of newly diagnosed insulin resistant-T2D patients. In humans, reduction in NLRP3 expression in adipose tissue is linked to decreased inflammation and improved insulin sensitivity in obese T2D patients. Recently, it was demonstrated that the lipolytic factor CGI-58 is an endogenous suppressor of NLRP3 activity in animal models. Therefore, the aim of this study was to evaluate CGI-58 and NLRP3 gene expressions in adipose tissue from individuals with different obesity degrees and their association with metabolic variables.

## Methodology

Subcutaneous adipose tissue was obtained from 35 individuals who undergone bariatric surgery. All individuals underwent a clinical and laboratory evaluation after signing an informed consent form. Fifteen individuals were classified as having morbid-obesity (body mass index (BMI) ≥ 40 kg/m2) and 20 as having moderate-obesity (BMI: 30.0 – 39.9 kg/m2). Gene expressions of CGI-58 and NLRP3 were evaluated using RT-qPCR technique.

## Results

The two analyzed groups were similar regarding homeostasis model assessment-IR (HOMA-IR), basal metabolic rate, and lipid and glycemic profile (all P >0.05). NLRP3 expression seems to be increased in morbid-obese patients as compared to the moderate-obesity group, although this difference did not reach formal statistical significance (1.44 (0.38 – 4.11) vs. 0.72 (0.31 – 3.60), P=0.058). In contrast, CGI-58 expression seems to be decreased in morbid-obese patients (0.47 (0.19 – 1.33) vs. 0.70 (0.28 – 2.15), P=0.070). Interestingly, we observed a positive correlation between NLRP3 expression and triglyceride levels (r=0.685, P=0.001). No significant correlations were observed between CGI-58 and NLRP3 expressions and HOMA-IR (P >0.05)

## Conclusions

Our preliminary results suggest that NLRP3 and CGI-58 gene expressions are different between morbid-obese and moderate-obese patients. Moreover, our data indicating that NLRP3 expression is correlated with triglycerides is in agreement with studies showing an effect of diet on NLRP3 regulation.

